# 4sUDRB-seq: measuring genomewide transcriptional elongation rates and initiation frequencies within cells

**DOI:** 10.1186/gb-2014-15-5-r69

**Published:** 2014-05-09

**Authors:** Gilad Fuchs, Yoav Voichek, Sima Benjamin, Shlomit Gilad, Ido Amit, Moshe Oren

**Affiliations:** 1 Department of Molecular Cell Biology, Weizmann Institute of Science, Rehovot 76100, Israel; 2 Department of Molecular Genetics, Weizmann Institute of Science, Rehovot 76100, Israel; 3 The Israel National Center for Personalized Medicine (INCPM), Weizmann Institute of Science, Rehovot 76100, Israel; 4 Department of Immunology, Weizmann Institute of Science, Rehovot 76100, Israel

## Abstract

Although transcriptional elongation by RNA polymerase II is coupled with many RNA-related processes, genomewide elongation rates remain unknown. We describe a method, called 4sUDRB-seq, based on reversible inhibition of transcription elongation coupled with tagging newly transcribed RNA with 4-thiouridine and high throughput sequencing to measure simultaneously with high confidence genome-wide transcription elongation rates in cells. We find that most genes are transcribed at about 3.5 Kb/min, with elongation rates varying between 2 Kb/min and 6 Kb/min. 4sUDRB-seq can facilitate genomewide exploration of the involvement of specific elongation factors in transcription and the contribution of deregulated transcription elongation to various pathologies.

## Background

Gene transcription is a multistep process consisting of recruitment of RNA polymerase II (Pol II) to promoters, transcription initiation, elongation, and termination. In addition to producing RNA polymers based on the DNA template, the Pol II holoenzyme also regulates numerous RNA processing events, including 5′ cap formation, splicing, polyadenylation, and RNA transport [1-7].

While historically most studies focused on understanding how promoter binding and transcription initiation are regulated, recent studies have shown that additional stages of the transcription process are also tightly regulated and are critical for gene activation. These studies have demonstrated that binding of Pol II to gene promoters is not sufficient for productive transcription. Instead, in the majority of genes Pol II is partly ‘paused’ 20 to 60 nt from the transcription start site (TSS), and several regulated steps are needed in order for Pol II to depart from the TSS and transcribe the rest of the gene [8-15].

The rate of Pol II movement through gene bodies has also been linked to various aspects of co-transcriptional RNA processing. For example, changes in transcription elongation rates can affect the outcome of the splicing machinery [7,16,17], and slow elongation by Pol II was shown to enhance the inclusion of specific weak exons [7]; in support of this notion, Pol II was found to accumulate at exons [18-20]. Likewise, the rate of elongation by Pol II has been linked to regulation of alternative polyadenylation [21]; indeed, Pol II accumulates at polyadenylation sites [9].

The importance of steps subsequent to transcriptional initiation is also underscored by the impact of misregulation of these steps on cellular and organismal viability. For example, many of the MLL gene translocation partners, thought to drive aggressive acute leukemia, are part of the super elongation complex (SEC). Leukemia-associated MLL fusion proteins relocalize the SEC to MLL target genes, bypassing normal transcriptional control and causing aberrant expression of those genes [22]. Similarly, excessive c-Myc was suggested to augment gene expression by increasing the release of paused Pol II and thus facilitating active elongation, thereby alleviating rate-limiting constraints on tumor cell growth and proliferation [23]. Furthermore, viruses utilize or modify transcription elongation-related processes to their benefit. For example, the influenza A NS1 protein comprises a histone-like sequence that can target the PAF1 elongation complex, enabling selective modulation of host cell gene expression and contributing to suppression of the antiviral response [24]. In addition, during active HIV-1 infection, viral transactivator of transcription (Tat) recruits the SEC to the HIV-1 long terminal repeat (LTR) to activate expression of the provirus in host cells [25-27]. Thus, better elucidation of the regulation of transcriptional elongation could contribute to the understanding of molecular mechanisms of disease, and even suggest novel therapeutic approaches.

Despite the documented links between elongation rates and numerous RNA-related functions, the actual elongation rates within cells remain under debate, with reported values ranging from 1 kilobase per minute (Kb/min) to 6 Kb/min [28]. In most cases, elongation rates of only a few genes at a time were measured; one study, utilizing Global Run-On sequencing (GRO-seq), was able to determine the rates for approximately 166 long genes upregulated in response to short treatment with physiological, non-toxic inducers [29]. Since the elongation rates of individual genes can be altered in a stimulus-dependent manner [29], it is important to measure the elongation rates of non-stimulated genes in order to derive more general understanding of the relationship between basal transcription elongation rates and steady state RNA processing and gene expression. Moreover, assessment of a large number of genes may provide more definitive information on the factors that affect transcription elongation rates. Here we describe a method that enables to easily measure simultaneously, in a single experiment, the steady state elongation rates of thousands of genes within live cells.

## Results and discussion

### Adaptation of the DRB assay for measuring elongation rates in short time scales by RNA sequencing

**Figure 1:**
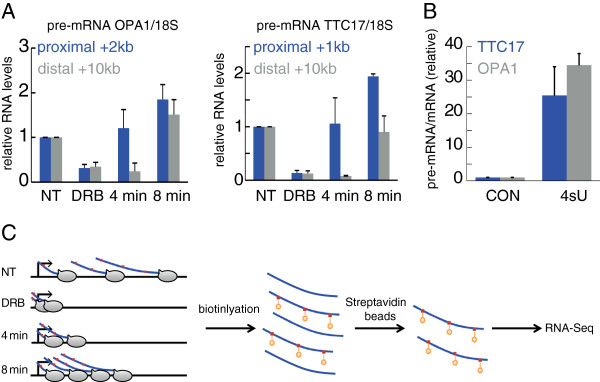
**Adaptation of the DRB assay for measuring elongation rates in short time scales by RNA sequencing. (A)** Analysis of *OPA1* and *TTC17* pre-RNA in HeLa cells, immediately after 3 h DRB treatment (DRB) and at the indicated time points after DRB removal. NT = non-treated. Pre-mRNA levels were determined by qRT-PCR, employing intronic primers. All values were normalized to *18S* RNA in the same sample. Levels in non treated cells were set as 1. Bars indicate averages of three independent experiments; error bars represent standard deviation. **(B)** Relative pre-mRNA/mRNA ratios in total RNA (CON) and 4sU-enriched RNA (4sU). HeLa cells were labeled for 8 min with 4sU (1mM). RNA was isolated, biotinylated, and enriched on streptavidin beads. qRT-PCR analysis of *OPA1* and *TTC17* pre-mRNA and mRNA was performed on the enriched RNA, as well as on total (non-enriched) RNA. The pre-mRNA/mRNA ratio for each gene in the CON sample was arbitrarily defined as 1.0. **(C)** Scheme of the timing of DRB and 4sU treatments in the different conditions. NT = non-treated, DRB = 3 h of DRB only, 4 min = 3 h of DRB and harvesting 4 min after DRB removal, 8 min = 3 h of DRB and harvesting 8 min after DRB removal. Red squares = 4sU, orange stars = biotin. Following the indicated treatments, isolated 4sU-labeled RNA was biotinylated and purified with magnetic streptavidin beads as described in [42] and subjected to high throughput sequencing.

Previously, Singh and Padgett [30] employed 5,6-dichlorobenzimidazole 1-β-d-ribofuranoside (DRB), which reversibly blocks transcription *in vivo*, combined with quantitative reverse transcriptase-PCR (qRT-PCR), to assess the elongation rates of several long human genes. We sought to adapt this method for simultaneous determination of the elongation rates of all DRB-sensitive genes. Since the median length of a human gene is approximately 24 Kb and typical elongation rates are estimated at a few Kb/min, measurements were performed 4 and 8 min after DRB removal. Pre-mRNA levels were quantified by qRT-PCR, employing primers specific for intronic sequences*.* Analysis of pre-mRNA levels in HeLa cells incubated with DRB for 3 h revealed that transcription of a proximal region of the *OPA1* gene, located 2 Kb downstream to the TSS, was recovered already within 4 min of DRB removal (Figure 1A). In contrast, full transcriptional recovery of a distal region of the same gene, located 10 Kb downstream to the TSS, occurred only 8 min after drug release. A similar trend was observed for *TTC17* (Figure 1A) and several other genes (data not shown). Hence, the DRB protocol can capture the progress of Pol II also in average-sized genes.

In order to obtain genomewide information on elongation rates, this protocol had to be adapted to RNA-seq. Nascent RNA constitutes only a very small fraction of the total RNA within cells; therefore, it is necessary to enrich it prior to sequencing. In principle, such enrichment is achievable by GRO-seq, which is becoming increasingly popular [29,31-33]. However, GRO-seq requires prior isolation of nuclei, which takes a relatively long time; this might introduce biases in short time scale measurements. We therefore added to the protocol a step of labeling nascent RNA *in vivo* with 4-thiouridine (4sU), which can be incorporated into intact cells [34,35], followed by specific purification of 4sU-containing RNA.

To test whether short 4sU labeling can indeed enrich for nascent RNA, HeLa cells were treated with 4sU for 8 min. 4sU-tagged RNA was biotinylated *in vitro*, purified on streptavidin beads and subjected to qRT-PCR analysis. Pre-mRNA/mature RNA ratios were calculated for *OPA1* and *TTC17* both in the 4sU-enriched fraction and in the total (non-enriched) RNA. Similar to a previous report [35], the short 4sU labeling yielded an approximately 20- to 35-fold enrichment of nascent RNA of these genes relative to the total RNA population (Figure 1B).

We next set out to determine the rate of movement of RNA Pol II on individual genes. To that end, we monitored the changes in nascent RNA abundance throughout those genes at different time points after resumption of RNA synthesis following DRB removal. HeLa cells were treated with DRB for 3 hours and harvested either 4 or 8 min following DRB removal. In both cases, the cells were pulsed with 4sU for the last 8 min before being harvested (Additional file 1). Cells were harvested in QIAzol lysis buffer directly on the culture dish. Next, 4sU-tagged nascent RNA from each sample was biotinylated and collected on streptavidin beads. This experimental procedure was performed separately in two biological repeats for the 4 min samples and four biological repeats for the 8 min samples. Finally, the RNA was subjected to deep sequencing (see Materials and Methods). The entire protocol, schematically illustrated in Figure 1C, was termed 4sUDRB-seq.

### Genomewide analysis of transcription elongation rates by 4sUDRB-seq

**Figure 2:**
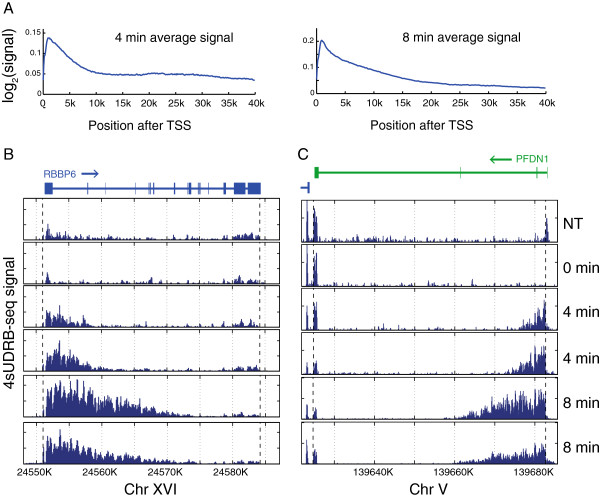
***In vivo *elongation rate analysis of thousands of genes simultaneously. (A)** Averaged distribution of 4sUDRB-seq reads over the different biological repeats in all genes longer than 20 Kb in RNA harvested 4 or 8 min after DRB removal. To minimize distortion of the data by residual mature mRNA, only reads within introns were considered and averaged. **(B, C)**. 4sUDRB-seq reads distribution along the *RBBP6* and *PFDN1* genes. Arrow marks the direction of transcription.

Given the high correlation between biological repeats (Additional file 2), we averaged the 4sUDRB-seq signals of the 4 and 8 min samples from the biological repeats over all genes longer than 20 Kb. We observed a distinct wave of transcription progression (Figure 2A). As expected, the nascent RNA reads in the 8 min sample extended much beyond the 4 min sample, relative to the TSS (marked as 0). Of note, the average distance traversed by Pol II between 4 and 8 minutes appears substantially longer than that attained in the first 4 min; this might imply either a slower elongation rate in the region immediately adjacent to the TSS followed by subsequent acceleration, or a short delay in resumption of transcription following DRB removal. A similar pattern was revealed by examination of single gene reads, showing a clear advance of nascent RNA synthesis between the 4 min and 8 min time points (Figure 2B, C).

The density of reads decreases gradually towards the advancing front of the transcription wave (Figure 2B, C). Hence, the exact border of the advancing transcription wave is not sharp and is hard to define. Furthermore, DRB does not enforce a complete transcription arrest, as evident by the presence of scattered low abundance reads throughout gene bodies (Figure 2B, C). Therefore, we developed an algorithm that determines the front border of the elongation wave for each gene at a given time point, taking into consideration the above observations. To avoid biases due to sequence properties, the pre-DRB release pattern was used for correction. Only genes with a primary transcript longer than 25 Kb were considered.

**Figure 3:**
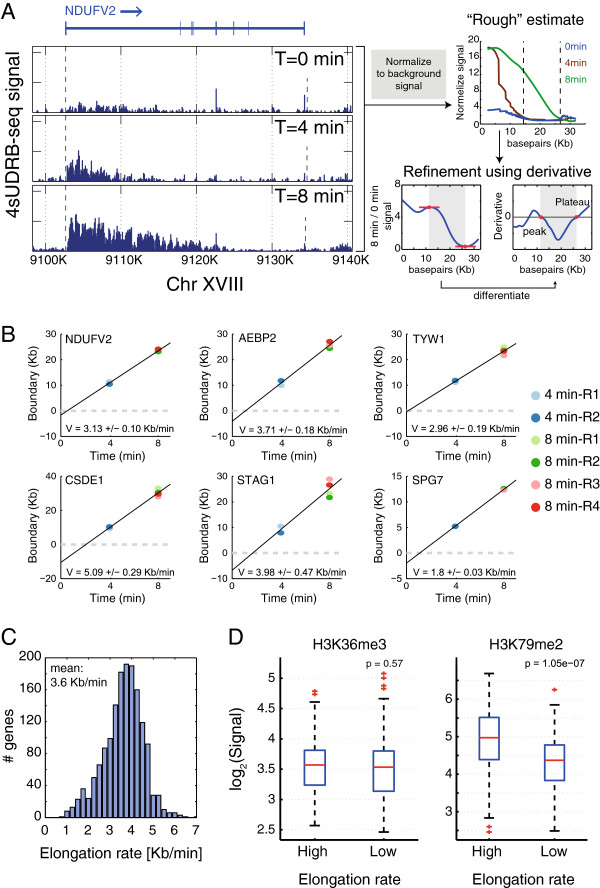
***In vivo *genomewide measurement of transcription elongation rates. (A)** Schematic representation of the algorithm used to infer elongation rates, exemplified for the *NDUFV2* gene. Left panel: 4sUDRB-seq data for 0/4/8 min after DRB release (similar to Figure 2B). Each signal was then corrected for the inferred background signal (right top panel), identifying for the 4 and 8 min samples the first position downstream to the TSS where the corrected signal is similar to that of the 0 time point; this location is designated by a vertical dashed line. To refine the boundary identification, we evaluated the background-corrected signals of the 4 and 8 min time points divided by the 0 time point signals and their derivatives. Refinement of the boundary position was then performed by monitoring the decreasing signal area in the vicinity of the rough boundary estimate, and determining the location between the closest peak and the point where the signal plateaus. **(B)** Linear fitting was performed on the averaged 4 and 8 min elongation boundaries as a function of time for the indicated genes. Elongation rates were defined by extracting the slope value of the linear fit (V). Confidence interval is indicated for each gene. **(C)** Distribution of measured elongation rates of all informative DRB-sensitive genes. **(D)** Box-whisker plot of log2 transformed H3K36me3 and H3K79me2 levels in genes that overlap with each modification in the 25% of genes with the highest calculated elongation rate and the 25% of genes with the lowest elongation rate. H3K79me2 is significantly higher in fast elongating genes (t-test, *P* = 1e-7). All data were adapted from ENCODE.

To determine the front end of the elongation wave for each transcript at 4 and 8 min after DRB release, we performed two sequential steps. First, we attempted to define the approximate position of the front end (‘rough’ estimate) by identifying the 5’-most point within the gene where the number of normalized reads is not any higher than at the 0 time point (Figure 3A). In the second step, we refined this estimate using the shape of the pattern. Basically, the algorithm looks at the derivative of the pattern while correcting for the background. As the abundance of reads decreases towards the front boundary of the elongation wave, the pattern derivative should become negative; however, at the exact location of the boundary it should become 0, as it is not affected anymore by transcription initiation due to DRB release (Figure 3A). Hence, the first position upstream to the region identified by the ‘rough’ estimate where the derivative became 0 was defined as the wave end point.

In some cases, the algorithm identified a clear front boundary for a particular gene in some of the experiments, but failed to do so in one or more of the other experiments (data not shown). Consequently, we calculated elongation rates only for genes that yielded distinct boundaries in at least two independent repeats of a given time point. To assess the reliability of our measurements, the confidence level of the elongation rate for each gene was calculated (Materials and Methods). As seen in Additional file 3, for most of the genes the confidence level was better than ± 0.5 Kb/min. For subsequent analysis, only genes with confidence levels better than ± 0.5 Kb/min were considered (Additional file 3). In addition, the estimated delay time, namely the time it takes transcription to reinitiate effectively following DRB removal, was also calculated; this was defined as the time interval missing in order for the distance between the TSS and the 4 min boundary to be equal to the distance between the 4 and 8 min boundaries, given the calculated elongation rate. This calculation is based on the assumption that the elongation rate is uniform throughout the gene. However, it remains formally possible that the rate is slower at the beginning of the gene, in which case the calculated delay times may represent overestimates.

Overall, we could measure with high confidence the elongation rates of 1,577 genes. The full list of elongation rates generated by this analysis is presented in Additional file 4. Examples of elongation rate measurements for several representative genes are shown in Figure 3B. As also seen in Figure 3C, while most genes are transcribed at a rate of approximately 3.5 Kb/min*,* actual transcription elongation rates vary between 2 Kb/min and 6 Kb/min. Overall, these rates are in the same range as those determined previously for a small number of specific genes [28,30].

To validate the rates deduced by 4sUDRB-seq, two genes with different calculated elongation rates (*RBM33* = 4.53 Kb/min, *CLCN3* = 2 Kb/min) were examined at higher temporal resolution, starting at 2 min after DRB release. Pre-mRNA was quantified by qRT-PCR with intron-derived primers. Reassuringly, the elongation rates determined by qRT-PCR (*RBM33* = 5 Kb/min, *CLCN3* = 2.7 Kb/min) were in reasonably good agreement with the rates determined by 4sUDRB-seq (Additional file 5A); the small differences might be due to the lower spatial resolution of qRT-PCR as compared to RNA-seq. Similar results were observed by measuring the elongation rates of those genes with different sets of primers (Additional file 5B).

Histone post-translational modifications (PTMs) were suggested to alter transcriptional elongation rates [36-38]. Hence, we asked whether the variance in elongation rates correlates with differences in a particular PTM. Interestingly 48% of genes in the upper quartile of genes with the highest elongation rate were found to be enriched in H3K79me2 within their gene body, compared to only 19% of genes from the slowest elongation rate quartile (Fisher’s exact test, *P* = 5.5e-18; data not shown). In contrast, no significant enrichment was observed for H3K36me3 (Fisher’s exact test, *P* = 0.99). Further analysis of genes that have H3K79me2 or H3K36me3 in their gene body confirmed that H3K79me2 is significantly higher in the fast elongation rate quartile (t-test, *P* = 1e-7), while H3K36me3 is relatively similar in both quartiles (t-test, *P* = 0.57) (Figure 3D). Since the average expression level was similar in both quartiles (t-test, *P* = 0.43, data not shown), the difference in H3K79me2 cannot simply be due to differences in expression. Further experiments will be required in order to determine whether H3K79me2 accelerates elongation or the high levels of this PTM are merely a consequence of the faster elongation. The fact that we did not detect a correlation between H3K36me3 and elongation rates is surprising, since such correlation was recently observed in response to a specific stimulus [29]. This might suggest that elongation during induced transcription utilizes a different set of histone PTMs than those associated with constitutive basal transcription.

### Calculation of genomewide relative transcription initiation frequencies by 4sUDRB-seq

**Figure 4:**
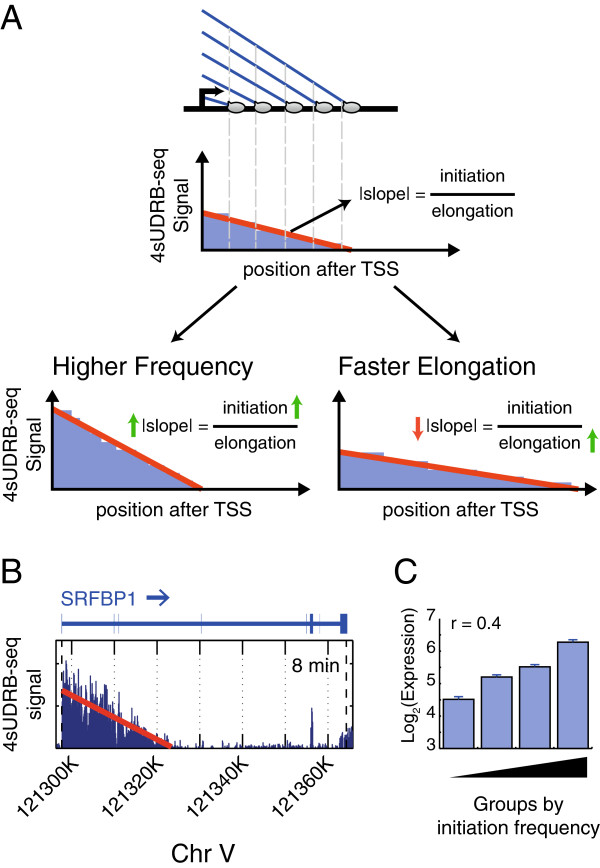
**Calculation of genomewide relative transcription initiation frequencies by 4sUDRB-seq. (A)** Schematic representation of the linear decrease in 4sUDRB-seq read intensity downstream to the TSS of a hypothetical gene and in a gene with similar elongation rate but higher initiation frequency (“Higher Frequency”) or in a gene with similar initiation frequency but faster elongation rate (“Faster Elongation”). **(B)** Analysis as in (A), exemplified for the 8 min time point of the *SRFBP1* gene, Red: linear fit of the signal upstream to the boundary found by our algorithm. **(C)** Average expression levels of four quartiles of genes binned by increasing initiation frequencies. r indicates Pearson correlation. Error bars = standard errors.

The 4sUDRB-seq reads are not uniformly distributed throughout gene bodies, decreasing gradually towards the 3′ end of the gene (see Figures 2 and 3A). The slope of the decreasing signal is dictated by the combined impact of the transcription initiation frequency and the elongation rate [39], “transcription initiation frequency” being defined here as the average frequency at which Pol II molecules transition from an initiation mode into an elongation mode and start moving into the gene body (Figure 4A) [40]. Thus, a greater number of such events occurring within a defined time window will result in a steeper slope (Figure 4A, Higher Frequency), whereas a faster elongation rate will tend to flatten the slope (Figure 4A, Faster Elongation). Hence, by measuring the slopes and elongation rates for each gene, the initiation frequencies can be estimated (Figure 4A, B). Using this approach, we calculated the relative initiation frequencies for all DRB-sensitive genes (Additional file 6). To exclude the possibility that our calculation is biased by an excessively high signal in the proximity of the TSS, owing to Pol II pausing, relative initiation frequencies were recalculated for all genes after excluding the 2 first 2 Kb immediately downstream to the TSS. As seen in Additional file 7, this did not affect significantly the deduced initiation frequencies. Next, we compared the calculated relative initiation frequencies to gene expression data in HeLa S3 cells (ENCODE). As seen in Figure 4C, a positive correlation was found between initiation frequencies and expression levels. Since gene expression levels are mostly determined by initiation frequencies [40], this correlation supports the reliability of the relative initiation frequencies deduced by 4sUDRB-seq.

## Conclusions

We report here that, by combining the reversible inhibitor DRB and 4sU tagging, transcription elongation rates and relative initiation frequencies within intact cells can be relatively easily measured on a genomewide scale. This method can be utilized in order to compare elongation rates between different regions within genes, different tissues, or different biological contexts such as differentiation and transformation. Moreover, by coupling 4sUDRB-seq with depletion of specific transcription-regulatory factors, one should be able to discriminate between factors that impact gene expression by modulating transcription initiation frequencies and those that affect the elongation step.

Note: while this manuscript was under revision, an essentially similar method was described by Veloso *et al.*[41].

## Materials and methods

### Cell culture and treatments

Human cervical carcinoma HeLa cells were grown in DMEM with 10% bovine serum supplemented with antibiotics and maintained at 37°C. 5,6-dichlorobenzimidazole 1-β-d-ribofuranoside (DRB) was purchased from Sigma (D1916), and used at a final concentration of 100μM. 4-thiouridine (4sU) was purchased from Sigma (T4509) and used at a final concentration of 1mM. In two of the NT, 0 and 8 min samples, cells were transfected with non-targeting siRNA (Dharmacon) 48 h before addition of DRB, employing the Dharmafect 1 reagent according to the manufacturer’s instructions.

### RNA purification and quantitative RT-PCR

For quantitative reverse transcriptase PCR (qRT-PCR) analysis, total RNA was extracted with the miRNeasy kit (Qiagen). Two microgram of each RNA sample was reverse transcribed with Moloney murine leukemia virus reverse transcriptase (Promega, Madison, WI, USA) and random hexamer primers (Applied Biosystems). Real-time PCR was done in a StepOne real-time PCR instrument (Applied Biosystems, Foster City, CA, USA) with Syber Green PCR supermix (Invitrogen).

### Biotinylation and purification of 4sU labeled RNA

Biotinylation and purification of 4sU labeled RNA was done as described in [42], with minor changes. A total of 100 to 180 μg RNA was used for the biotinylation reaction. 4sU-labeled RNA was biotinylated using EZ-Link Biotin-HPDP (Pierce), dissolved in dimethylformamide (DMF) at a concentration of 1 mg/mL and stored at -80°C. Biotinylation was done in labeling buffer (10 mM Tris pH 7.4, 1 mM EDTA) and 0.2 mg/mL Biotin-HPDP for 2 h with rotation at 25°C. Unbound Biotin-HPDP was removed by chloroform/isoamylalcohol (24:1) extraction using MaXtract (high density) tubes (Qiagen). RNA was precipitated at 20,000 *g* for 20 min at 4°C with a 1:10 volume of 5M NaCl and an equal volume of isopropanol. The pellet was washed with an equal volume of 75% ethanol and precipitated again at 20,000 *g* for 10 min at 4°C. The pellet was left to dry followed by resuspension in 100 μL RNase-free water. Biotinylated RNA was captured using Dynabeads MyOne Streptavidin T1 beads (Invitrogen). Biotinylated RNA was incubated with 100 μL Dynabeads with rotation for 15 min at 25°C. Beads were magnetically fixed and washed with 1× Dynabeads washing buffer. RNA-4sU was eluted with 100 μL of freshly prepared 100 mM dithiothreitol (DTT), and cleaned on RNeasy MinElute Spin columns (Qiagen).

### Preparation and sequencing of 4sU labeled RNA

cDNA libraries were prepared with Illumina TruSeq RNA sample preparation v2 kit according to the manufacturer’s instructions but without the polyA isolation stage. Of note, strand specificity is not achieved with this kit. Random hexamers were used for reverse transcription. Libraries were pooled and sequenced on an Illumina HiSeq 2500 system using the paired-end 50 mode.

### Read mapping

All reads were aligned to the human reference genome (hg19, GRCh37) using the bowtie 2 aligner with default parameters (only setting -N 1) [43]. We considered only reads mapped uniquely to the genome and not to chrUn_gl000220 (rRNA) for further analysis. For each experiment we constructed a genome-wide profile of the signal per disjoint 100 bp adjacent bins. The sum of each experiment was normalized to be 10^6^.

### Gene annotations

All gene annotations were taken from the hg19 RefGene tab. of the UCSC browser.

### Boundary detection algorithm

Only genes longer than 25 Kb and with at least 20 intron bins with positive numerical values were included in the boundary detection algorithm. In addition, in cases where a gene has multiple variants, we consider only the longest one.

#### ‘Rough estimate’

For each of the filtered genes from the different 4 min or 8 min samples we extracted only the profile data of introns, in order to avoid the confounding effects of reads derived from contaminating mature mRNA. In order to increase the reliability of the signal used for normalization, and to reduce the effects of variability between the 0 min samples due to low read numbers, the profile of the 0 time point was created by averaging four different biological repeats of the 0 min samples. Next, the intron data were smoothed using cubic spline (smoothing parameter - 10^-5^).

To normalize each gene in each experiment (0, 4, and 8 min) by the background signal, we performed the following procedure. First, for each bin of the gene we estimated the background signal downstream to this bin up to the 3’ end of the gene. This background read distribution was built out of a negative binomial signal. To estimate the average of the background distribution we took the most abundant value. The most abundant value was identified by building a histogram of the data and selecting the bin with the highest count. In cases where we had fewer than 10 values downstream to the last point with positive signals we used the background estimate of the upstream bin.

We next compared the 4 and 8 min normalized profiles to the 0 time point. For each of the 4 and 8 min samples, we chose the first bin downstream the TSS + 2.5 Kb (defined arbitrarily) where the sample’s signal is not higher than the 0 time point at the same position (up to 10%). This bin was defined as the ‘rough estimate’ of the boundary. If the first position (TSS + 2.5 Kb) was identified or no position was identified no boundary was determined.

#### Refinement using profile derivative

For each gene we took the profile of the introns and as in the ‘rough estimate’, we used cubic spline with the same parameter. Before smoothing the 0 time point bins, we added the value 0.01 to each bin (according to the ‘Rule of Succession’) in order to prevent cases where data are normalized to 0 time points and results in dividing by 0. Then we divided the 4 and 8 min gene profiles by the 0 time point profile and obtained the derivative by subtracting each adjacent bin.

We looked for the local minimums of the derivative using the N.Yoder peak finder (mathworks.com) upstream the ‘rough estimate’ boundary; we considered only derivatives smaller than -0.01. We took the most downstream minimum relative to the ‘rough estimate’ boundary. Next, we identified the most downstream bin relative to the minimum (up to 2 Kb downstream to the ‘rough estimate’) where the derivative was close to 0 (> = -0.002) and defined it as the ‘refined estimate’. If no such bin was found we adhered to the ‘rough’ estimate.

#### Calculating elongation rate and rate confidence interval

In order to calculate the elongation rates, only genes with for which clear elongation boundaries were identified by the algorithm in both of the 4 min samples and in at least three of the four 8 min samples were included. Next, genes in which the average of the 8 min boundaries was lower than that of the 4 min boundaries were excluded. Since we had four biological repeats for the 8 min samples, we used modified Thompson tau outlier method in order to eliminate a maximum of one outlier. Next, the elongation rate was calculated by linearly fitting the averaged 4 and 8 min boundaries as a function of time. The slope of the linear fit was defined as the elongation rate. Next, the 50% confidence interval of the slope was defined as the confidence interval of the elongation rate. Only genes with confidence intervals better than 0.5 Kb/min were retained. The slope was also used to define the delay time, which was defined as being equal to the slope intersection with the time axis. The elongation rate was determined only for genes with delay times between -1 and 4 min.

### Processing of ENCODE data

***H3K36me3/H3K79me2*** data of Hela-S3 were derived from the ENCODE project [44]. Peak calling for H3K36me3 and H3K79me2 was done as follows: for each gene the 20 Kb downstream to the TSS were binned into 100 bp bins, and peak calling was done using N.Yoder peak finder (mathworks.com). Only genes with at least one peak were selected. Next, in order to get an enrichment level for each gene we averaged for each modification the signal for all bins.

***RNA-seq*** data of Hela-S3 were also from the ENCODE project (Caltech RnaSeq Helas3 200SigRep1V4) [44]. Expression per gene was the average for the signal on all exons.

### Calculating relative initiation frequency

For a constant initiation frequency *I* and a constant RNA polymerase velocity  *V*, the relative amount of polymerases that have moved downstream to location *x* in the gene within *T*  minutes (*T* = 8 in our case) from DRB removal will be (with delay time *D*):

$$\mathrm{Signal}\left(x\right)=I\left(T-D-\frac{x}{V}\right)=I\left(T-D\right)-x\left(\frac{I}{V}\right)$$

To infer the initiation frequency we calculated the best linear fit of the intron signal as a function of Kb downstream to the TSS: *ax* + *b*. If we denote by B the 8 minutes boundary, we get:

$$\mathrm{aB}+b\approx 0\left(\mathrm{Not}\;\mathrm{exactly}\;\mathrm{zero}\;\mathrm{due}\;\mathrm{to}\;\mathrm{noise}\right)$$

Adding the previous equality we get:

$$I\approx \frac{\mathrm{bV}}{B}$$

The intron signal was derived from each 8 min sample, divided by the averaged signal from the 0 min samples plus 1 (as pseudocounts to prevent dividing by 0). This calculation was performed separately for all four biological repeats and outliers were excluded using the modified Thompson Tau outlier method. Relative initiation frequencies were determined by averaging the rates from the biological repeats.

## Primers

Primers used in this study: OPA1 mRNA F 5′-TTTTTACCTCAGGTTCTCCGGA, OPA1 mRNA R 5′CACGATCTGTTGCTCTAAACGC; OPA1+2 Kb F 5′ ACCATGGATGCCATTGAGTCA, OPA1+2 Kb R 5′TGTGCCATCACCAGGAGACAT; OPA1+10 Kb F 5′TCTGTTCCATGATGAGCTGTGG, OPA1+10 Kb R 5′CCTGGTCCTTCCTGAATCTTTG; TTC17 mRNA F 5′ATCAAAGCCAAGGTGCCCT, TTC17 mRNA R 5′GGACTGATGTCTTTGCTCTCCA; TTC17+1 Kb F 5′ TCAGAGGCGAGACTGCTTTTC, TTC17+1 Kb R 5′GCATTTACAAAACGCAGGCA; TTC17+10 Kb F 5′ TCCAGCCTCAGACACCACTTT, TTC17+10 Kb R 5′ TGGTTTGAAGAACATCCCGAG; RBM33+5 Kb F 5′TTCCACATCTTCCTGGCACA, RBM33+5 Kb R 5′ACACAGGTGAATCATGTGGAAT; RBM33+10 Kb F 5′GAACTCAGCCTCTGTGCTGT, RBM33+10 Kb R 5′ACAATGTGATGAGGGCTGGG; RBM33+14.2 Kb F 5′TCCTCTCTGCCAGTCTGTGA, RBM33+14.2 Kb R 5′GAAGGACTGGCATCTGGCAT; RBM33+20 Kb F 5′CCATGATTTGGAAAAGTGTGACGA, RBM33+20 Kb R 5′TGTGACATGCTAAAAAGTTAGAGAC; CLCN3+4 Kb F 5′ACAGCATCCCTCTTGAGGAAAA, CLCN3+4 Kb R 5′GGCCCTACAGCTTTCAGTAGA; CLCN3+9.3 Kb F 5′ACGTTCTACAATGGCAGACAGA, CLCN3+9.3 Kb R 5′GCTTCGCTGACCCACTTACT; CLCN3+20 Kb F 5′ACAGGAAGGGAGAGCCAAGA, CLCN3+20 Kb R 5′AGCTGCACTCATGAACAGTCA; 18S F 5′CGCCGCTAGAGGTGAAATTCT, 18S R 5′CATTCTTGGCAAATGCTTTCG.

### Data availability

Raw data have been deposited into GEO with accession number: GSE57116.

## Competing interests

The authors declare that they have no competing interests.

## Authors' contributions

GF, YV, IA and MO conceived the study and designed the experiments. GF performed the experiments. YV analyzed the data. SB and SG generated the libraries for sequencing and performed the sequencing. GF, YV and MO wrote the paper. All authors have read and approved the manuscript for publication.

## Supplementary Material

Additional file 1 **Related to Figure 1: Schematic representation of biological samples for 4sUDRB-seq.** HeLa cells were either not treated (NT) or treated with DRB for 3 hours and harvested either 4 or 8 min following DRB removal. In both cases, the cells were pulsed with 4sU for the last 8 min before being harvested. Click here for file

Additional file 2 **Related to Figure 2: Correlation between biological repeats.** Pearson correlation between the average signals over introns for all genes longer than 10Kb. The specific treatment (NT, 0 min, 4 min, 8 min) and the sample number (1,2,3,4) are indicated for each repeat sample. Click here for file

Additional file 3 **Related to Figure 3: Confidence interval distribution.** Confidence interval distribution for all genes for which elongation boundaries were successfully detected by our algorithm in the 4 and 8 min samples. Genes left to the red dotted line were taken for further analysis as described in the text. Click here for file

Additional file 4 **Related to Figure 4: List of elongation rates and their confidence intervals.** Only genes with confidence intervals better than ±0.5 Kb were included. Click here for file

Additional file 5 **Related to Figure 5: Validation of transcription elongation rates. (A, B)** qRT-PCR analysis of *RBM33* and *CLCN3* pre-mRNA in HeLa cells, without DRB treatment (NT) and at the indicated time points after DRB removal. To simulate the experimental conditions of the DRB-seq experiment, 4sU was added to all cultures 8 min before harvesting, although subsequent biotinylation and purification were not performed because the use of intronic primers in the qRT-PCR procedure already selects for pre-mRNA. All values were normalized to *18S* RNA in the same sample. Bars indicate averages of data from duplicate qPCR reactions; error bars represent standard deviation. (A) and (B) are derived from two independent experiments, using different qPCR primer pairs. Click here for file

Additional file 6 **Related to Figure 6: List of relative transcription initiation frequencies.** Relative initiation frequencies were calculated by averaging slope values of four biological repeats of the 8 min samples. Outliers were excluded. Click here for file

Additional file 7 **Related to Figure 7: Correlation of relative initiation frequencies calculated either with or without inclusion of the first 2Kb of genes.** Scatter plot of relative initiation frequencies calculated for full transcripts (x-axis) vs. relative initiation frequencies calculated after excluding the first 2 Kb of each transcript (y-axis). Click here for file
