# Supplementary figures and images for: 4sUDRB-seq: measuring genomewide transcriptional elongation rates and initiation frequencies within cells
Source: Genome Biol. 2014 May 9;15(5):R69. doi: 10.1186/gb-2014-15-5-r69 (PMC4072947; doi:10.1186/gb-2014-15-5-r69)

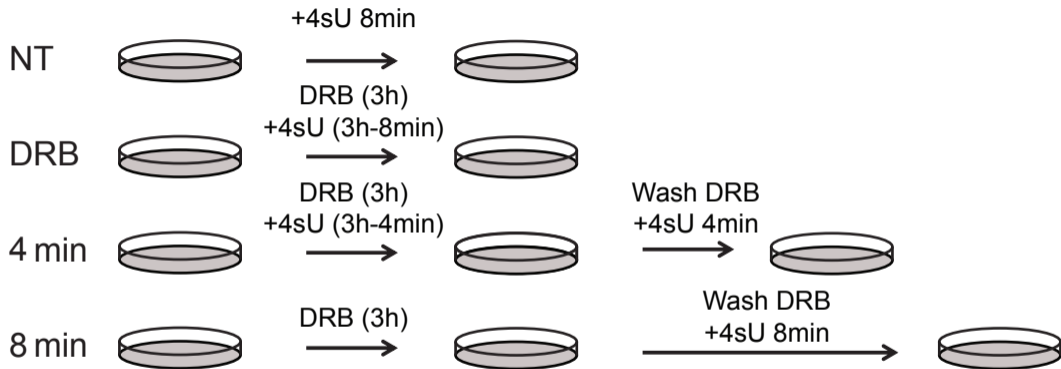

**Additional file 1**

Supplement: Additional file 1 — Related to Figure 1: Schematic representation of biological samples for 4sUDRB-seq. HeLa cells were either not treated (NT) or treated with DRB for 3 hours and harvested either 4 or 8 min following DRB removal. In both cases, the cells were pulsed with 4sU for the last 8 min before being harvested. [file gb-2014-15-5-r69-S1.pdf]

Correlation of average signal over introns for all genes

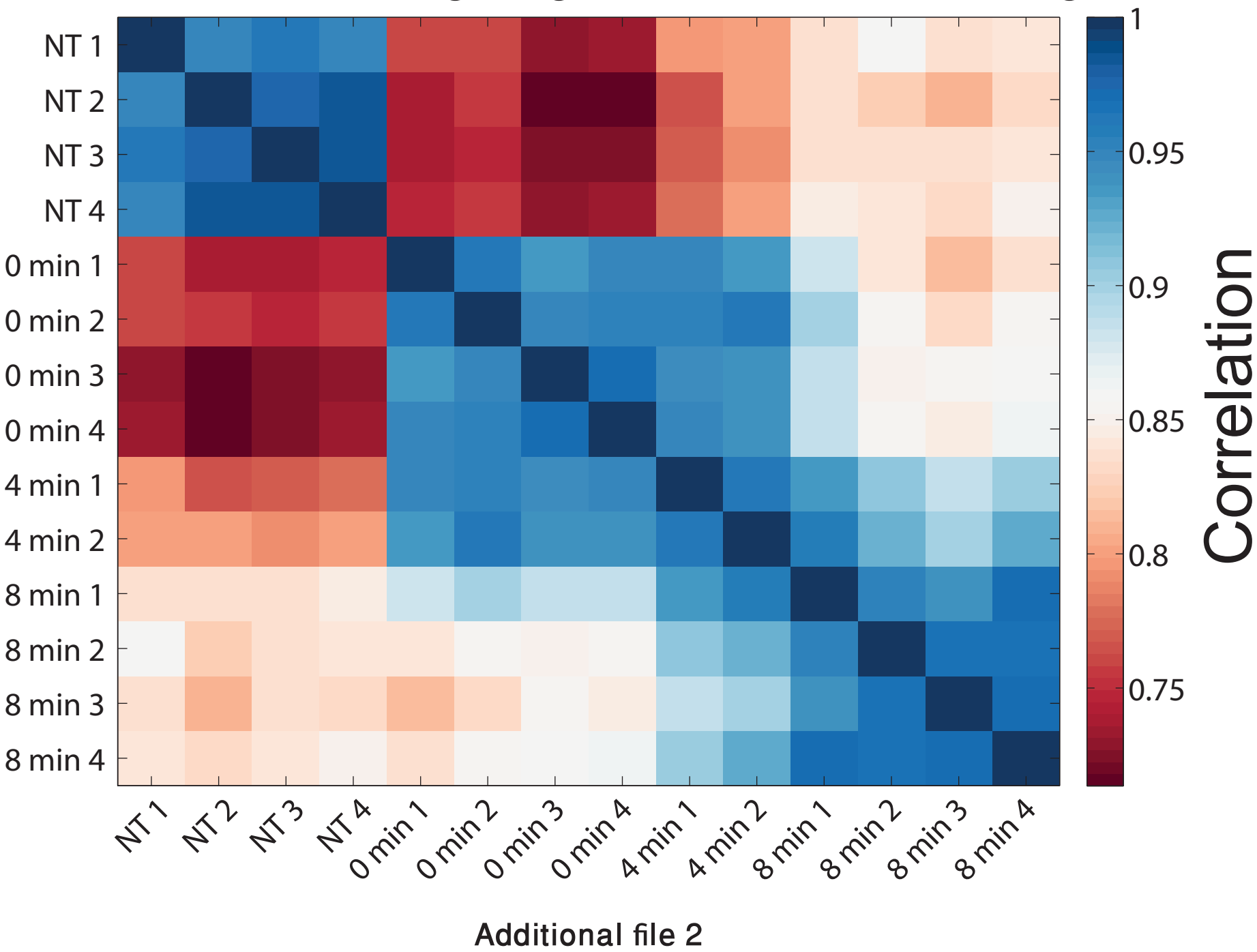

Supplement: Additional file 2 — Related to Figure 2: Correlation between biological repeats. Pearson correlation between the average signals over introns for all genes longer than 10Kb. The specific treatment (NT, 0 min, 4 min, 8 min) and the sample number (1,2,3,4) are indicated for each repeat sample. [file gb-2014-15-5-r69-S2.pdf]

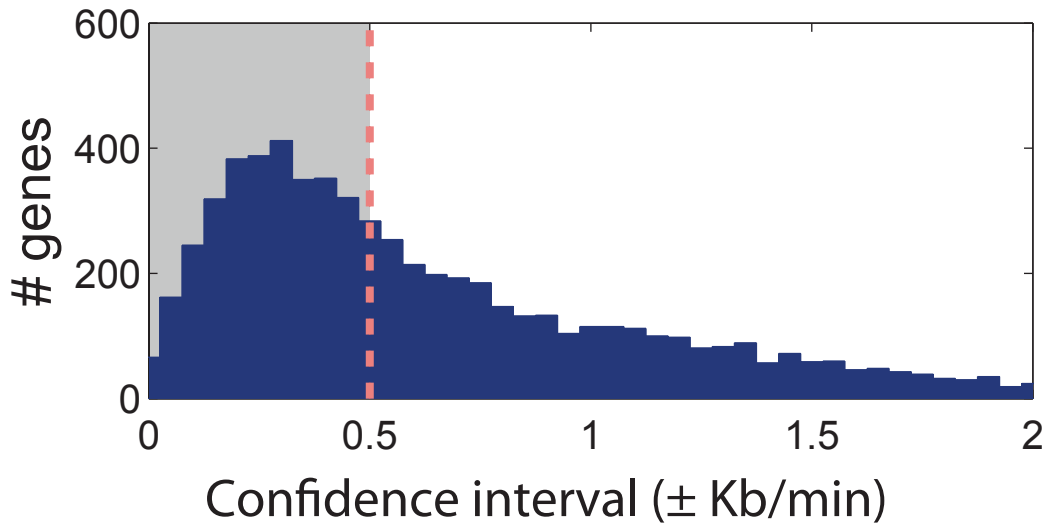

Additional file 3

Supplement: Additional file 3 — Related to Figure 3: Confidence interval distribution. Confidence interval distribution for all genes for which elongation boundaries were successfully detected by our algorithm in the 4 and 8 min samples. Genes left to the red dotted line were taken for further analysis as described in the text. [file gb-2014-15-5-r69-S3.pdf]

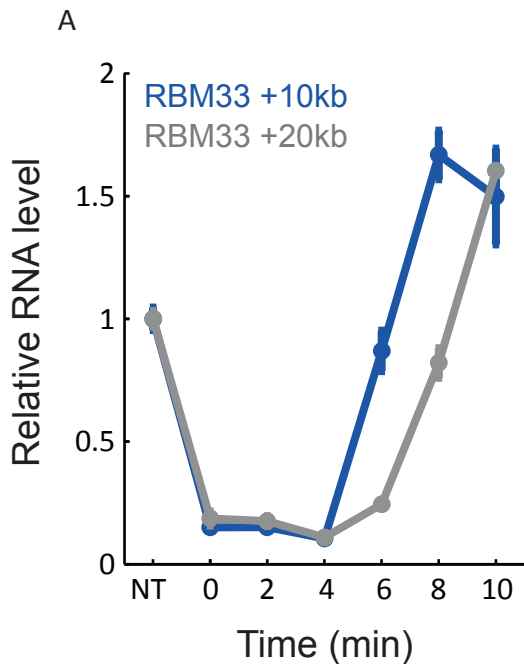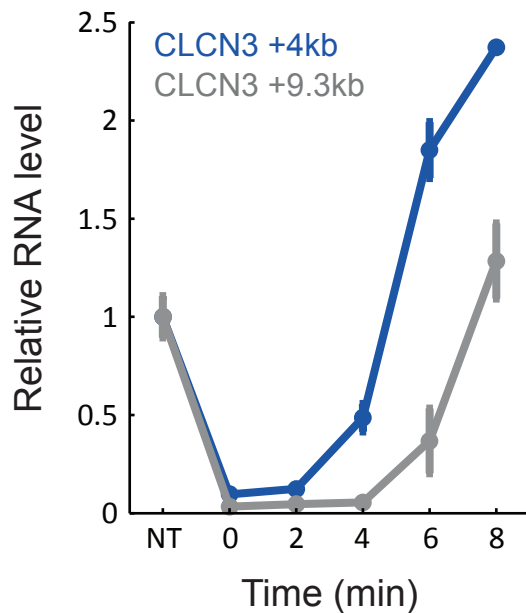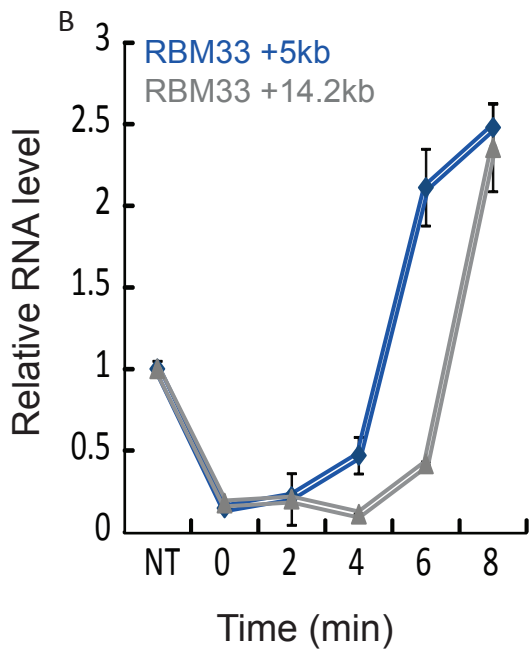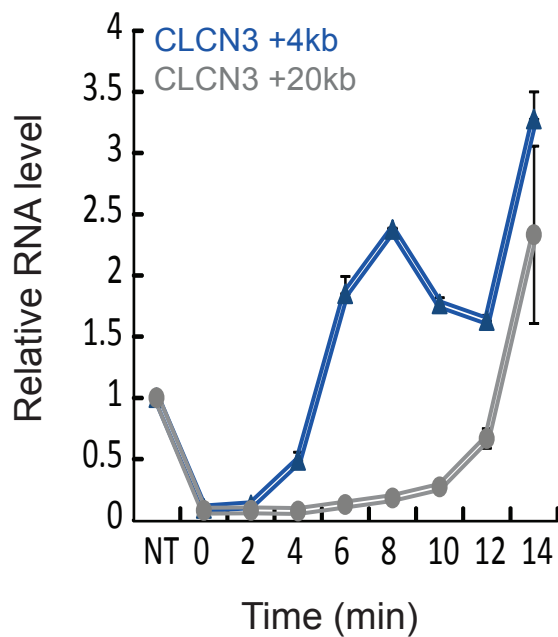

Supplement: Additional file 5 — Related to Figure 5: Validation of transcription elongation rates. (A, B) qRT-PCR analysis of RBM33 and CLCN3 pre-mRNA in HeLa cells, without DRB treatment (NT) and at the indicated time points after DRB removal. To simulate the experimental conditions of the DRB-seq experiment, 4sU was added to all cultures 8 min before harvesting, although subsequent biotinylation and purification were not performed because the use of intronic primers in the qRT-PCR procedure already selects for pre-mRNA. All values were normalized to 18S RNA in the same sample. Bars indicate averages of data from duplicate qPCR reactions; error bars represent standard deviation. (A) and (B) are derived from two independent experiments, using different qPCR primer pairs. [file gb-2014-15-5-r69-S5.pdf]

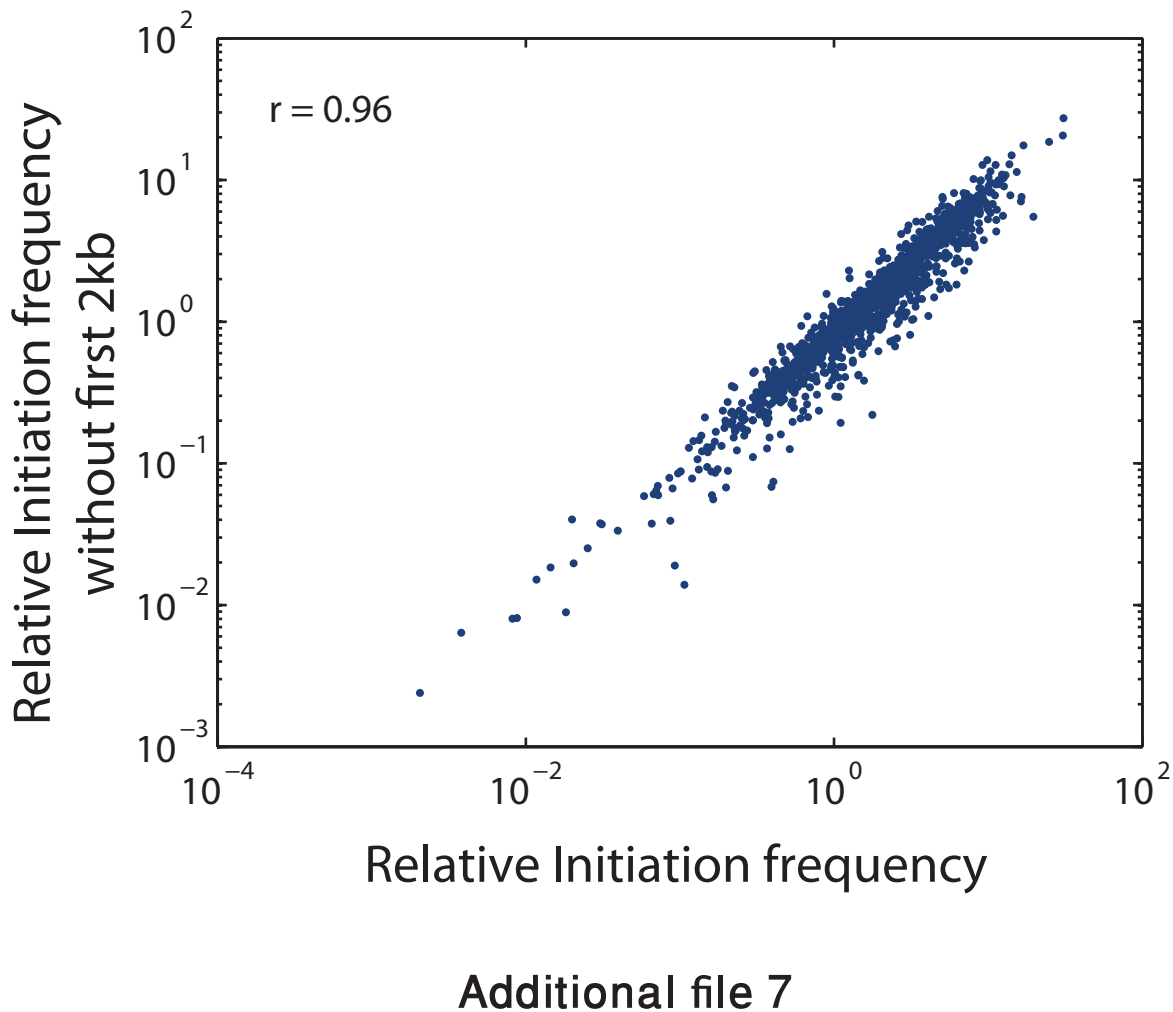

Supplement: Additional file 7 — Related to Figure 7: Correlation of relative initiation frequencies calculated either with or without inclusion of the first 2Kb of genes. Scatter plot of relative initiation frequencies calculated for full transcripts (x-axis) vs. relative initiation frequencies calculated after excluding the first 2 Kb of each transcript (y-axis). [file gb-2014-15-5-r69-S7.pdf]
